# Comparison of brain microstructure alterations on diffusion kurtosis imaging among Alzheimer’s disease, mild cognitive impairment, and cognitively normal individuals

**DOI:** 10.3389/fnagi.2022.919143

**Published:** 2022-08-12

**Authors:** Xiaoqi Chu, Peng Wu, Hongting Yan, Xuejing Chen, Liting Fan, Zheng Wu, Chunmei Tao, Yue Ma, Yu Fu, Yunchu Guo, Yang Dong, Chao Yang, Yusong Ge

**Affiliations:** ^1^Department of Neurology, Second Affiliated Hospital of Dalian Medical University, Dalian, China; ^2^School of Medicine, Nankai University, Tianjin, China; ^3^Department of Radiology, Second Affiliated Hospital of Dalian Medical University, Dalian, China; ^4^Department of Radiology, First Affiliated Hospital of Dalian Medical University, Dalian, China

**Keywords:** Alzheimer’s disease, mild cognitive impairment, diffusion kurtosis imaging, neuropsychological test, brain microstructure alterations

## Abstract

**Objective:**

Our study aimed to explore the differences in brain microstructure in patients with Alzheimer’s disease (AD) and with mild cognitive impairment (MCI) and in individuals with normal cognition using diffusion kurtosis imaging (DKI) to identify a potential non-invasive biomarker of AD.

**Materials and methods:**

A total of 61 subjects were included in our study, including 20 subjects diagnosed with AD, 21 patients diagnosed with amnestic MCI, and 20 cognitively normal individuals. We acquired magnetic resonance imaging (MRI) scans, and DKI images were processed. Twelve regions of interest were drawn, and various parameters were measured and analyzed using SPSS version 11.0 software.

**Results:**

Comparative analysis showed that differences in brain regions in terms of mean diffusion (MD) and mean kurtosis (MK) between groups were the most marked. Precuneus MD, temporal MK, precuneus MK, and hippocampal MK were significantly correlated with neuropsychological test scores. Hippocampal MK showed the strongest correlation with the medial temporal lobe atrophy score (*r* = −0.510), and precuneus MD had the strongest correlation with the Koedam score (*r* = 0.463). The receiver operating curve analysis revealed that hippocampal MK exhibited better diagnostic efficacy than precuneus MD for comparisons between any group pair.

**Conclusion:**

DKI is capable of detecting differences in brain microstructure between patients with AD, patients with MCI, and cognitively normal individuals. Moreover, it compensates for the deficiencies of conventional MRI in detecting pathological changes in microstructure before the appearance of macroscopic atrophy. Hippocampus MK was the most sensitive single parameter map for differentiating patients with AD, patients with MCI, and cognitively normal individuals.

## Introduction

With the increase in the number of older adults, the incidence of neurodegenerative diseases is also rising, for which aging is the main contributing factor (Kritsilis et al., 2018). Alzheimer’s disease (AD) is the most common neurodegenerative disease and the leading cause of dementia, affecting approximately 3% of patients aged 65 to 75 years, 17% of patients aged 75 to 84 years, and 32% of patients aged over 84 years old (Abdelnour et al., 2016; Weller and Budson, 2018; Babulal et al., 2019; Alzheimer’s Association, 2020). Owing to genetic factors and women’s longer life expectancy, the number of female patients is two times that of male patients (Soria et al., 2019). AD has an insidious onset and manifests as progressive cognitive impairment and behavioral abnormalities. As the disease progresses, patients gradually become unable to take care of themselves in daily life, which can overwhelm caregivers and society in general. Mild cognitive impairment (MCI) refers to the stage of cognitive impairment that is not sufficient to be diagnosed as dementia; the National Institute of Aging and the Alzheimer’s Association first issued the diagnostic criteria for MCI in 2011 based on the 1984 diagnostic standards. This shifted the diagnosis time point of AD forward and enabled early clinical interventions to be realized (Albert et al., 2011; Roberts and Knopman, 2013).

Approximately, 50 million people worldwide are affected by AD, and the annual cost of dementia in the United States alone is as high as US$600 billion. It is estimated that the number of patients with dementia will increase rapidly in the next few decades, and the magnitude of this increase is likely to be greater in low- and middle-income countries, presenting severe challenges to both individual families and government departments (Mantzavinos and Alexiou, 2017; Lane et al., 2018; Rusek et al., 2019). Unfortunately, there is currently no effective method to prevent or delay the progression of AD. Pathological changes in AD can occur as early as 20 years before symptoms appear (Alzheimer’s Association, 2020), and considerable information is missed because research has primarily focused on cases that are at the clinical stage of AD. If AD can be detected early, as yet unidentified pathogenic factors could be examined, it could contribute to the development of more effective treatments; moreover, early applications of existing drugs could be trialed in the preclinical stage. In addition, other interventions, such as nutrition, exercise, cognitive training, and social interactions, can be planned as early as possible in the preclinical stage when patient cooperation and treatment effects are likely to be better. Therefore, early detection, regular monitoring in the preclinical phase, and early treatment are vital for accurate clinical diagnoses and effective treatments.

Diffusion kurtosis imaging (DKI) is an imaging technology based on the theory of non-Gaussian motion of water molecules in tissues (Hui et al., 2012; Bonilha et al., 2015; Zhang et al., 2015; Abdalla et al., 2020). The biological structure of the nervous system is so complex that changes in the myelin sheath, axons, cell membranes, organelles, and proteins can all lead to pathology. Based on the non-Gaussian motion of water molecules and the fourth-order three-dimensional tensor mode, DKI overcomes the limitations of conventional magnetic resonance imaging (MRI) techniques, such as T1, T2, and T2-fluid attenuate inversion recovery imaging (T2-FLAIR), which cannot quantitatively analyze tissues, and the constraints of diffusion tensor imaging (DTI) on the true motion patterns of water molecules, which can better describe the differences between and changes in complex microstructures (Arab et al., 2018). Falangola et al. (2013) were the first to apply DKI to a clinical study of patients with AD and MCI and showed that all kurtosis parameter values in the two groups were significantly lower than those in the normal cognition group; moreover, the parameter values of the AD group were the lowest, which indicated that significant changes in kurtosis can distinguish patients with MCI from patients with AD. Gong et al. (2013) used DKI to analyze changes in kurtosis parameters in the cerebral lobes and revealed that gray matter damage in patients with AD spreads from the temporal lobe to the parieto-occipital cortex and frontal lobe. Other studies that compared deep and cortical gray matter and analyzed the hippocampus in detail further confirmed that DKI can detect microstructural changes in patients with AD and abnormalities in areas that cannot be identified using DTI (Wang et al., 2015; Chen et al., 2017; Gong et al., 2017; Song et al., 2019). Thus, DKI parameters can distinguish cognitively normal individuals from patients with MCI and AD and may enable the identification of a reliable biomarker for AD diagnosis and treatment.

As an indispensable element of clinical diagnosis, neurological tests play an important role in characterizing the cognitive changes in AD and the degree of cognitive impairment. AD and MCI diagnoses are based on a patient’s symptoms, neuropsychological test results, hippocampal and parietal lobe atrophy, and the exclusion of other causes of cognitive impairment. In our study, brain imaging data were acquired using DKI technology in patients with AD, patients with MCI, and cognitively normal individuals. Various parameters were compared among the three groups to explore the differences in DKI parameters among patients with AD, patients with MCI, and cognitively normal individuals, and correlation analyses among the imaging parameters, neuropsychological test results, and hippocampal and parietal atrophy were performed. In addition, we used the area under the curve (AUC) of the receiver operating characteristic (ROC) curve to evaluate the diagnostic efficacy of each parameter and identify a potential non-invasive biomarker for AD.

## Materials and methods

### Subjects

Our study was approved by the Ethics Committee of the Second Hospital of Dalian Medical University, and informed consent was obtained from each subject or guardian (Ethics Review of The Second Hospital of Dalian Medical University, No. 016, December 31, 2021). Sixty-one sex- and age-matched subjects (20 patients with AD, 21 patients with amnestic MCI, and 20 cognitively normal individuals) were enrolled. A total of seven patients were excluded, three of whom refused to participate in the study and four did not meet the inclusion criteria (three for high Fazekas grading and one for claustrophobia). AD participants were diagnosed by two experienced neurologists according to the standard criteria of the National Institute of Neurological and Communicative Disorders and Stroke/AD and Related Disorders Association for clinically probable AD. Amnestic patients with MCI were diagnosed according to the criteria in the study by Petersen (2004). Detailed inclusion criteria are shown in Table 1. In addition to the criteria in Table 1, all three groups met the following conditions: (1) MRI showed a maximum of two cerebral infarction lesions with a diameter of more than 2 cm to ensure the minimal influence of cerebrovascular events and excluded other brain injuries; (2) subjects had no consciousness disorder, aphasia or dysarthria, history of mental illness, severe organ dysfunction, or contraindications to MRI.

**TABLE 1:** Enrollment criteria for subjects.

Inclusion criteria		NC (*N* = 20)	MCI (*N* = 21)	AD (*N* = 20)
Age-bracket		60–90	60–90	60–90
Cognitive impairment		×	√	√
Daily life and work		Not affected	Not affected	Affected
Neuropsychological scores	MMSE	>26	≥21 and ≤26	≥10 and ≤26
	CDR	0	0.5	≥0.5
	ADL	20	≤22	>22
	HIS	≤4	≤4	≤4
Fazekas grading	Age ≤70 years	≤Grade 1	≤Grade 1	≤Grade 1
	Age >70 years	≤Grade 2	≤Grade 2	≤Grade 2

NC, normal cognition; MCI, mild cognitive impairment; AD, Alzheimer’s disease; MMSE, mini-mental state examination; CDR, clinical dementia rating scale; ADL, activity of daily living scale; HIS, Hachinski ischemic score.

### Neuropsychological assessment and evaluation of brain atrophy

All subjects were evaluated by two trained neuropsychological assessors using a series of scales to evaluate the cognitive domains. Comprehensive scales comprised the mini-mental state examination (MMSE), the Montreal cognitive assessment scale (MoCA), the Hasegawa dementia scale revised (HDSR), and the clinical dementia rating scale (CDR). Other scales comprised the digit span test (DST), the verbal fluency test (VFT), the frontal assessment battery (FAB), the trail making test A (TMTA), the Rey auditory verbal learning test (RAVLT; consisting of a total of four scores for immediate recall, short-term delayed recall, long-term delayed recall, and recognition), the neuropsychiatric inventory-questionnaire (NPI-Q; the NPI-Q1 score reflected the patient’s score, and the NPI-Q2 score represented the caregiver’s score), the Hamilton anxiety scale (HAMA), and the Hamilton depression scale (HAMD). The activity of the daily living scale (ADL) was used to assess whether patients had the ability to take care of themselves, which distinguished patients with AD from patients with MCI.

The medial temporal lobe atrophy (MTA) scale was used to evaluate hippocampal atrophy. Grade 1 indicated widening of the choroidal fissure only; Grade 2 indicated the accompaniment of the enlargement of the temporal angle of the lateral ventricle; Grade 3 indicated a moderate reduction in hippocampal volume; Grade 4 indicated a severe decrease in hippocampal volume. The Koedam scale was used to evaluate parietal lobe atrophy. Grade 0 indicated a closed posterior cingulate and parieto-occipital sulcus and closed sulci of the parietal lobes and precuneus; Grade 1 indicated a mild widening of the posterior cingulate and parieto-occipital sulci, with mild atrophy of the parietal lobes and precuneus; Grade 2 indicated a substantial widening of the posterior cingulate and parieto-occipital sulci, with substantial atrophy of the parietal lobes and precuneus; Grade 3 indicated end-stage atrophy, evident widening of both sulci, and knife-blade atrophy of the parietal lobes and precuneus.

### Magnetic resonance imaging data acquisition and processing

Before the MRI examination, the contraindications to MRI of each patient were checked, and both the purpose of the examination and the scanning time were informed in detail. During the scan, patients were instructed to relax and remain as still as possible. Because patients with cognitive impairment, especially those with dementia, were easily disturbed by the external conditions of the examination, they were given sound insulation headsets to minimize the noise of the scanner. A 3.0T Siemens Verio MRI scanner was used to acquire the data with a 32-channel head coil. Parameters of MRI acquisition are shown in Table 2. The *b* = 0 image represented the anatomical phase without orientation. When b reached 1000 or 2000, the number of motion probing gradient (MPG) directions was 15.

**TABLE 2:** MRI acquisition parameters.

Parameter	T_1_WI	T_2_WI	T_2–_FLAIR	DKI
TR (ms)	250	4000	9000	2200
TE (ms)	2.46	99	108	103
TI (ms)	–	–	2500	–
Thi (mm)	5.5	5.5	5.5	5
Slice	20	20	20	24
FOV (mm × mm)	230 × 230	230 × 201	230 × 201	220 × 220
Matrix size	320 × 256	320 × 320	320 × 240	192 × 192
Voxel size (mm)	0.7 × 0.7 × 5.5	0.4 × 0.4 × 5.5	0.7 × 0.7 × 5.5	0.6 × 0.6 × 5
Flip angle (°)	70	150	150	–
Acquisition time (s)	66	70	128	278
b (s/mm^2^)	–	–	–	0,1000,2000
Directions	–	–	–	30

T_1_WI, T1-weighted imaging; T_2_WI, T2-weighted imaging; T_2–_FLAIR, T2-fluid attenuate inversion recovery imaging; DKI, diffusion kurtosis imaging; TR, repetition time; TE, echo delay time; TI, inversion time; Thi, slice thickness; FOV, field of view.

To obtain diffusion kurtosis maps, the logarithm of signal attenuation following the diffusion sensitization sequence is described as follows, which represents a cumulant expansion for the diffusion magnetic resonance signal:

ln[S(b)] = ln(S_0_)-bD_*app*_ + $\frac{1}{6}$b^2^D^2^_*app*_K_*app*_ + O(b^3^),

where S(b) refers to signal intensity, D_*app*_ refers to the diffusion coefficient, and K_*app*_ refers to diffusional kurtosis. When *K* = 0, Gaussian formalism is restored (Jensen and Helpern, 2010).

**FIGURE 1:**
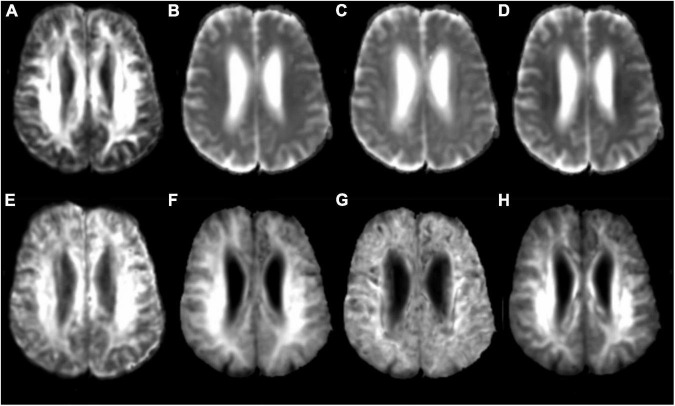
Diffusion kurtosis imaging (DKI) parameter image. **(A)** FA: fractional anisotropy; **(B)** MD: mean diffusion; **(C)** Da: axial diffusion; **(D)** Dr: radial diffusion; **(E)** FAK: fractional anisotropy of kurtosis; **(F)** MK: mean kurtosis; **(G)** Ka: axial kurtosis; **(H)** Kr: radial kurtosis.

**FIGURE 2:**
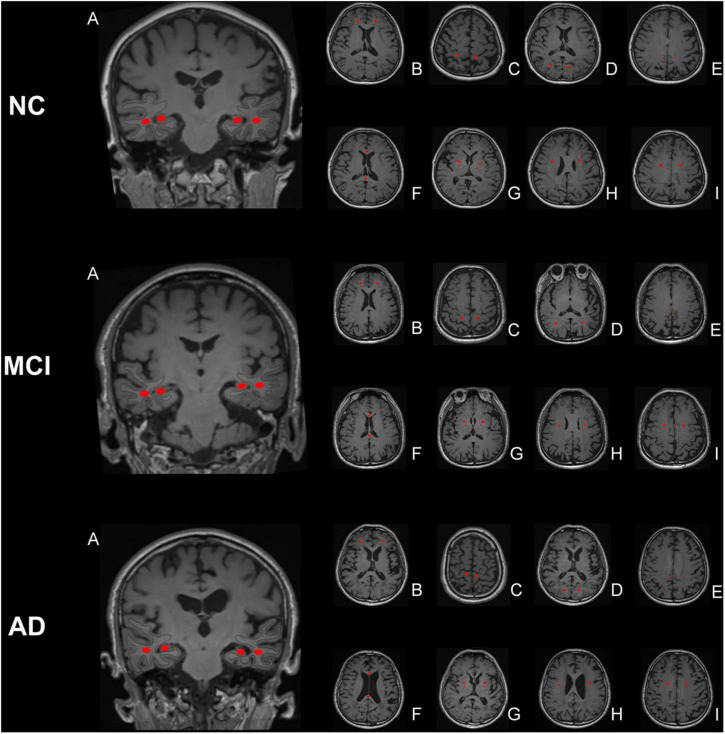
Region of interest (ROI) placement diagram. The selected ROIs are shown on the T1 maps. **(A)** temporal lobe and hippocampus; **(B)** frontal lobe; **(C)** parietal lobe; **(D)** occipital lobe; **(E)** precuneus; **(F)** splenium of the corpus callosum and genu of the corpus callosum; **(G)** posterior limb of the internal capsule and lenticular nucleus; **(H)** corona radiata; **(I)** centrum semiovale. The central part of the largest layer and the upper and lower adjacent layers of the structure were selected, and the same sized ROIs were drawn in the symmetrical region of the bilateral lobes, avoiding the interference areas, such as the cerebrospinal fluid and blood vessels.

The original DKI data (DICOM format) were converted into analyzable data (NIFIT format) and then processed by the FSL software of Oxford University (FMRIB Software Library)^1^ using the “dcm2niigui” toolkit in the MRIcron software.^2^ Preprocessing, e.g., head motion and eddy current correction, was performed. The DKI image data acquired were post-processed using the Diffusion Kurtosis Estimator (DKE) software,^3^ and various parameter maps were obtained, including fractional anisotropy (FA) from DKI, mean diffusion (MD) from DKI, axial diffusion (Da) from DKI, radial diffusion (Dr) from DKI, fractional anisotropy of kurtosis (FAK), mean kurtosis (MK), axial kurtosis (Ka), and radial kurtosis (Kr) (Figure 1). Considering the possibility of misidentifying brain regions with the use of automatic whole-brain analysis, manual region of interest (ROI) analysis was chosen for the follow-up work. Using the MK parameter map as the main parameter map, ROIs were manually drawn by means of the MRIcron software. ROIs, which are the areas related to clinical diagnosis, the areas relevant to cognitive impairment, and the areas of previous research (Struyfs et al., 2015; Chen et al., 2017; Gong et al., 2017), respectively, are described below and shown in Figure 2. All ROIs were drawn by two neuroradiologists (Peng Wu and Yang Dong) and checked by a third senior expert (Chao Yang) to ensure correct anatomical position. Peng Wu was responsible for the ROI drawing of the subcortical white matter of the frontal lobe, parietal lobe, temporal lobe, occipital lobe and precuneus, hippocampus, and Yang Dong was responsible for the ROI drawing of the splenium of the corpus callosum, genu of the corpus callosum, posterior limb of the internal capsule, lenticular nucleus, corona radiata, and centrum semiovale. The central part of the largest layer and the upper and lower adjacent layers of the structure were selected, and the same sized ROIs were also drawn in the symmetrical region of the bilateral lobes, avoiding the interference areas, such as the cerebrospinal fluid and blood vessels. The measurement was repeated three times. Measurements of the left and right lobes were taken together as a whole, and the average value of the three repeated measurements was used for the final statistical analysis.

### Statistical analysis

Statistical analysis was performed using the SPSS version 11.0 software. The normal distribution of data was confirmed using the normality test (PP plot method). Continuous data were compared among the three groups using a one-way analysis of variance (ANOVA) or analysis of covariance (ANCOVA) with age and sex included as confounding factors. For multiple comparisons between groups, the least significant difference method was used for groups with uniform variance, and the Tamhane method was used for groups with unequal variance. Furthermore, FDR correction was also used for multiple comparisons of DKI parameters. An independent sample *t*-test was used to compare continuous data between two groups. Pearson’s correlation analysis was used to analyze correlations between two continuous variables. For graded data, the overall differences among the three groups were compared using the Kruskal–Wallis test, comparisons between groups were carried out using the Mann–Whitney *U*-test, and the correlation analysis was conducted using Spearman’s correlation. Chi-square tests were used to compare categorical data between groups, and different statistical results were used according to the number of cases and theoretical frequency. If the number of cases was greater than 40, and the theoretical frequency was greater than five, the result of the normal chi-square test or the maximum likelihood/Fisher’s exact probability was used. The AUC was used to evaluate the diagnostic efficacy of each parameter. A *p* < 0.05 indicated statistical significance. For the Mann–Whitney *U*-test, pairwise comparisons among the three groups were carried out three times, and the significance level (*p*-value) was adjusted to 0.017.

## Results

### General characteristics, neuropsychological tests, and brain atrophy evaluation

Apart from disease duration, there were no significant differences in age, sex, educational level, history of hypertension, history of diabetes, or history of smoking among the three groups (*p* > 0.05; Table 3).

**TABLE 3:** General characteristics.

		NC (*N* = 20)	MCI (*N* = 21)	AD (*N* = 20)	χ^2^/t/F	*p*
Age (years)		73.30 ± 9.02	71.67 ± 6.65	75.35 ± 8.80	1.036	0.361
Duration of disease		–	1.74 ± 0.44	2.78 ± 0.60^#^	6.382	<0.001
Sex [*n* (%)]	Male	4 (20)	9 (42.9)	6 (30)	2.514	0.285
	Female	16 (80)	12 (57.1)	14 (70)		
Duration of education [*n* (%)]	Primary school	5 (25)	2 (9.5)	1 (5)	4.097	0.664
	Junior high	9 (45)	13 (61.9)	13 (65)		
	Senior high	4 (20)	4 (19)	4 (20)		
	University	2 (10)	2 (9.5)	2 (10)		
Hypertension [n (%)]	Yes	11 (55)	12 (57.1)	11 (55)	0.026	0.987
	No	9 (45)	9 (42.9)	9 (45)		
Diabetes Mellitus [n (%)]	Yes	11 (55)	7 (33.3)	8 (40)	2.050	0.359
	No	9 (45)	14 (66.7)	12 (60)		
History of smoking [n (%)]	Yes	7 (35)	5 (23.8)	5 (25)	0.760	0.684
	No	13 (65)	16 (76.2)	15 (75)		

NC, normal cognition; MCI, mild cognitive impairment; AD, Alzheimer’s disease. ^#^*p* < 0.05 vs. MCI group.

All neuropsychological test scores were significantly different among the three groups (*p* < 0.05). The scores of the MMSE, MoCA, HDSR, DST, VFT, FAB, and RAVLT of the AD group were lower than those of the normal cognition group, whereas the CDR, TMTA, NPI-Q1, NPI-Q2, HAMA, HAMD, and ADL scores were higher than those of the normal cognition group (*p* < 0.05). The scores of the MMSE, MoCA, HDSR, VFT, FAB, and RAVLT of the MCI group were lower than those of the normal cognition group, whereas the HAMA and HAMD scores were higher than those of the normal cognition group (*p* < 0.05). The scores of the MMSE, MoCA, HDSR, DST, VFT, FAB, and RAVLT of the AD group were lower than those of the MCI group, whereas the CDR, TMTA, NPI-Q1, NPI-Q2, and ADL scores were higher than those of the MCI group (*p* < 0.05; Table 4).

**TABLE 4:** Neuropsychological test results.

		NC (*N* = 20)	MCI (*N* = 21)	AD (*N* = 20)	*F*	*p*
MMSE		28.10 ± 0.79	25.10 ± 1.48*	15.60 ± 3.07*^#^	211.247	<0.001
MoCA		27.70 ± 0.66	21.10 ± 3.08*	10.35 ± 4.10*^#^	172.309	<0.001
HDSR		28.60 ± 0.50	24.76 ± 1.09*	12.60 ± 3.63*^#^	290.460	<0.001
CDR		0.00 ± 0.00	0.50 ± 0.00	1.50 ± 0.58*^#^	104.224	<0.001
DST		12.25 ± 0.72	11.43 ± 1.75	9.60 ± 0.60*^#^	27.516	<0.001
VFT		37.95 ± 3.40	30.71 ± 5.98*	19.65 ± 7.93*^#^	46.331	<0.001
FAB		17.70 ± 0.57	15.95 ± 2.13*	9.70 ± 3.21*^#^	70.175	<0.001
TMTA (s)		58.56 ± 7.64	72.93 ± 22.85	132.73 ± 42.84*^#^	38.806	<0.001
RAVLT	RAVLT immediate	38.80 ± 1.70	30.52 ± 5.51*	10.00 ± 3.84*^#^	271.120	<0.001
	RAVLT short	10.25 ± 0.97	4.90 ± 2.63*	0.05 ± 0.22*^#^	192.932	<0.001
	RAVLT long	9.00 ± 1.17	3.10 ± 2.70*	0.00 ± 0.00*^#^	141.308	<0.001
	RAVLT recognition	13.60 ± 1.35	10.57 ± 2.01*	0.65 ± 2.00*^#^	277.294	<0.001
NPI-Q	NPI-Q1	0.00 ± 0.00	1.48 ± 5.27	11.65 ± 16.78*^#^	7.943	0.001
	NPI-Q2	0.00 ± 0.00	1.00 ± 3.00	7.20 ± 9.69*^#^	9.033	<0.001
HAMA		1.55 ± 4.84	11.29 ± 8.30*	9.70 ± 7.21*	11.403	<0.001
HAMD		1.30 ± 4.00	11.19 ± 10.68*	13.55 ± 7.57*	13.385	<0.001
ADL		20.00 ± 0.00	20.24 ± 0.63	31.95 ± 8.34*^#^	40.996	<0.001

NC, normal cognition; MCI, mild cognitive impairment; AD, Alzheimer’s disease; MMSE, mini-mental state examination; MoCA, Montreal cognitive assessment scale; HDSR, Hasegawa dementia scale revised; CDR, clinical dementia rating scale; DST, digit span test; VFT, verbal fluency test; FAB, frontal assessment battery; TMTA, trail making test A; RAVLT, Rey auditory verbal learning test (immediate recall/short-term delayed recall/long-term delayed recall/recognition); NPI-Q, neuropsychiatric inventory-questionnaire (NPI-Q1: the score of patient, NPI-Q2: the score of caregiver); HAMA, Hamilton anxiety scale; HAMD, Hamilton depression scale; ADL, activity of daily living scale. *p < 0.05 vs. NC group; *^#^p* < 0.05 vs. MCI group.

The MTA and Koedam scores were significantly different among the three groups (*p* < 0.05). Compared with the normal cognition group, the MTA and Koedam scores of the AD group were significantly higher (*p* < 0.05). In comparison with the MCI group, the MTA score of the AD group was significantly higher (*p* < 0.05; Table 5).

**TABLE 5:** Evaluation of hippocampal atrophy and parietal lobe atrophy [n (%)].

		NC (*N* = 20)	MCI (*N* = 21)	AD (*N* = 20)*^#^	H	*p*
MTA score	Grade 0	16 (80)	7 (33.3)	0 (0)	35.885	<0.001
	Grade 1	2 (10)	9 (42.9)	1 (5)		
	Grade 2	2 (10)	5 (23.8)	14 (70)		
	Grade 3	0 (0)	0 (0)	5 (25)		
Koedam score	Grade 0	14 (70)	6 (28.6)	4 (20)	15.537	<0.001
	Grade 1	6 (30)	15 (71.4)	9 (45)		
	Grade 2	0 (0)	0 (0)	7 (35)		

NC, normal cognition; MCI, mild cognitive impairment; AD, Alzheimer’s disease, MTA, medial temporal lobe atrophy. *p < 0.05 vs. NC group in MTA score and Koedam score; ^#^*p* < 0.05 vs. MCI group in MTA score.

### Abnormalities in diffusion kurtosis imaging parameters

Various DKI parameters of all ROIs were significantly different among the three groups (*p* < 0.05).

Significant differences in DKI parameters for each ROI between the AD and normal cognition groups were as follows (*p* < 0.05): frontal lobe: MD and MK; parietal lobe: MD, Dr, and FAK; occipital lobe: MD and Dr; temporal lobe: FA, MD, Dr, FAK, MK, and Kr; precuneus: MD, Da, Dr, MK, Ka, and Kr; hippocampus: MD, Da, Dr, FAK, MK, Ka, and Kr; splenium of the corpus callosum: MD and MK; genu of the corpus callosum: MD, Dr, MK, and Kr; the posterior limb of the internal capsule: MD, Da, and MK; lenticular nucleus: Da and Kr; corona radiata: MD and MK; centrum semiovale: FA, MD, Dr, FAK, and MK.

Significant differences in DKI parameters for each ROI between the MCI and normal cognition groups were as follows (*p* < 0.05): temporal lobe: FA, FAK, and MK; precuneus: MD and Da; hippocampus: Dr, FAK, MK, and Kr; splenium of the corpus callosum: MK; genu of the corpus callosum: Dr and MK; lenticular nucleus: Kr.

**FIGURE 3:**
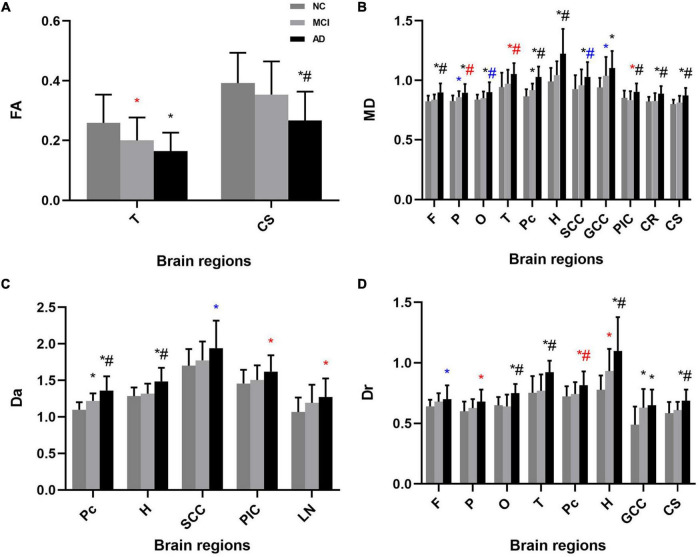
Mean (± standard deviation) diffusivity for each ROI for each study group in both one-way analysis of variance (ANOVA) and analysis of covariance (ANCOVA). **(A)** FA, **(B)** MD, **(C)** Da, and **(D)** Dr showed significant differences between groups. AD patients had higher diffusivity in most ROIs than the MCI and normal cognition groups. NC, normal cognition; MCI, mild cognitive impairment; AD, Alzheimer’s disease; FA, fractional anisotropy; MD, mean diffusion; Da, axial diffusion; Dr, radial diffusion; F, frontal lobe; P, parietal lobe; O, occipital lobe; T, temporal lobe; Pc, precuneus; H, hippocampus; SCC, splenium of the corpus callosum; GCC, genu of the corpus callosum; PIC, posterior limb of the internal capsule; LN, lenticular nucleus; CR, coronal radiata; CS, centrum semiovale. *p < 0.05 vs. NC group in ANOVA, ^#^*p* < 0.05 vs. MCI group in ANOVA; *p < 0.05 vs. NC group in ANCOVA, ^#^*p* < 0.05 vs. MCI group in ANCOVA; *p < 0.05 vs. NC group in both analysis, ^#^*p* < 0.05 vs. MCI group in both analysis.

**FIGURE 4:**
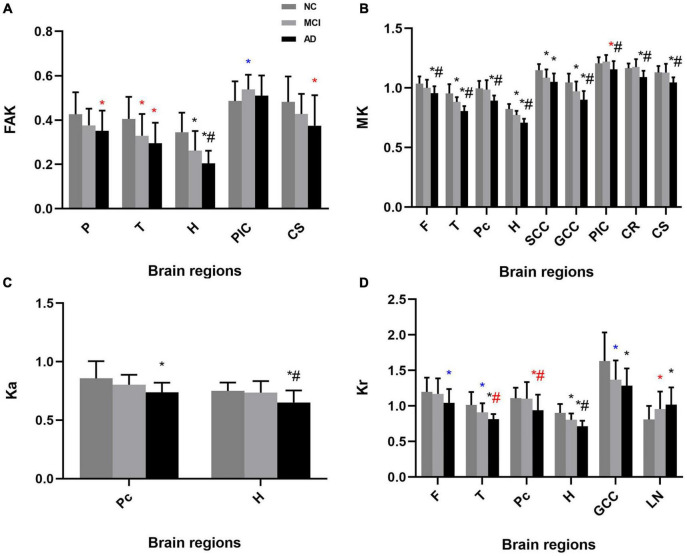
Mean (± standard deviation) kurtosis for each ROI for each study group in both one-way analysis of variance (ANOVA) and analysis of covariance (ANCOVA). **(A)** FAK, **(B)** MK, **(C)** Ka, and **(D)** Kr showed significant differences between groups. AD patients had lower kurtosis in most ROIs than the MCI and normal cognition groups. NC, normal cognition; MCI, mild cognitive impairment; AD, Alzheimer’s disease; FAK, fractional anisotropy of kurtosis; MK, mean kurtosis; Ka, axial kurtosis; Kr, radial kurtosis; F, frontal lobe; P, parietal lobe; O, occipital lobe; T, temporal lobe; Pc, precuneus; H, hippocampus; SCC, splenium of the corpus callosum; GCC, genu of the corpus callosum; PIC, posterior limb of the internal capsule; LN, lenticular nucleus; CR, coronal radiata; CS, centrum semiovale. *p < 0.05 vs. NC group in ANOVA, ^#^*p* < 0.05 vs. MCI group in ANOVA; *p < 0.05 vs. NC group in ANCOVA, ^#^*p* < 0.05 vs. MCI group in ANCOVA; *p < 0.05 vs. NC group in both analysis, ^#^*p* < 0.05 vs. MCI group in both analysis.

Significant differences in DKI parameters for each ROI between the MCI and AD groups were as follows (*p* < 0.05): frontal lobe: MD and MK; parietal lobe: MD; occipital lobe: Dr; temporal lobe: MD, Dr, MK, and Kr; precuneus: MD, Da, Dr, MK, and Kr; hippocampus: MD, Da, Dr, FAK, MK, Ka, and Kr; genu of the corpus callosum: MK; the posterior limb of the internal capsule: MD and MK; corona radiata: MD and MK; centrum semiovale: FA, MD, Dr, and MK (Figures 3, 4).

Considering the effect of age and sex, and the possibility of false positives (Hyatt et al., 2020), we also performed ANCOVA with age and sex as covariates and FDR correction for the comparisons of DKI parameters.

The results of ANCOVA indicated that several parameters were related to age and sex. Parameters with significant changes were as follows (p < 0.05): frontal lobe: Dr, Kr; parietal lobe: MD, Dr, FAK; occipital lobe: MD; temporal lobe: FA, MD, FAK, Kr; precuneus: Dr, Kr; hippocampus: Dr; splenium of the corpus callosum: MD, Da; genu of the corpus callosum: MD, Kr; the posterior limb of the internal capsule: MD, Da, FAK, MK; lenticular nucleus: Da, Kr; centrum semiovale: FAK (Figures 3, 4 and Supplementary Table 1).

The results of FDR correction in comparisons of DKI parameters are shown in Supplementary Table 2.

Parameters showing significant changes between the AD and normal cognition groups were as follows (p (FDR) <0.05): frontal lobe: MD, MK, and Kr; parietal lobe: MD, Dr, and FAK; occipital lobe: MD and Dr; temporal lobe: FA, MD, Dr, FAK, MK, and Kr; precuneus: MD, Da, Dr, MK, Ka, and Kr; hippocampus: MD, Da, Dr, FAK, MK, Ka, and Kr; splenium of the corpus callosum: MD, Da, and MK; genu of the corpus callosum: MD, Dr, MK, and Kr; the posterior limb of the internal capsule: MD, Da, and MK; lenticular nucleus: Da, Kr; corona radiata: MD, Da, and MK; centrum semiovale: FA, MD, Dr, FAK, and MK.

Parameters showing significant changes between the MCI and normal cognition groups were as follows [p (FDR) <0.05]: temporal lobe: MK; precuneus: Da; hippocampus: MK.

Parameters showing significant changes between the AD and MCI groups were as follows [p (FDR) <0.05]: frontal lobe: MD; occipital lobe: Dr; temporal lobe: Dr, MK, and Kr; precuneus: MD, Da, and MK; hippocampus: MD, Da, MK, Ka, and Kr; genu of the corpus callosum: MK; the posterior limb of the internal capsule: MD and MK; corona radiata: MD and MK; centrum semiovale: FA, MD, Dr, and MK.

### Correlation analysis of diffusion kurtosis imaging parameters

As shown above, the analysis of DKI parameters revealed a total of 11 ROIs that differed significantly for MD and 9 ROIs that differed significantly for MK. MD and MK were more sensitive indicators than other parameters for the evaluation of pathological changes in brain tissue in patients with cognitive impairment. Therefore, our follow-up correlation analysis was conducted for MD and MK.

Neuropsychological test scores were correlated with all brain regions for the analysis of MD, which was focused on the frontal lobe, parietal lobe, occipital lobe, temporal lobe, precuneus, hippocampus, genu of the corpus callosum, posterior limb of the internal capsule, corona radiata, and centrum semiovale (Tables 6, 7). For MK, correlations were primarily distributed in the frontal lobe, temporal lobe, precuneus, hippocampus, splenium of the corpus callosum, genu of the corpus callosum, posterior limb of the internal capsule, corona radiata, and centrum semiovale (Tables 8, 9).

**TABLE 6:** Pearson’s correlation analysis between MD and neuropsychological test.

MD	F	P	O	T	Pc	H
MMSE	−0.493**	−0.348**	−0.466**	−0.373**	−0.722**	−0.504**
MoCA	−0.432**	−0.328**	−0.445**	−0.365**	−0.687**	−0.477**
HDSR	−0.436**	−0.360**	−0.431**	−0.365**	−0.713**	−0.528**
CDR	0.336**	0.326*	0.402**	0.355**	0.714**	0.539**
DST	−0.273*	−0.359**	−0.345**	−0.500**	−0.481**	−0.456**
VFT	−0.345**	−0.282*	−0.312*	−0.451**	−0.542**	−0.288*
FAB	−0.371**	−0.386**	−0.405**	−0.323*	−0.616**	−0.432**
TMTA	0.363**	0.378**	–	0.258*	0.470**	0.333**
RAVLT immediate	−0.505**	−0.395**	−0.451**	−0.389**	−0.700**	−0.501**
RAVLT short	−0.492**	−0.447**	−0.390**	−0.359**	−0.680**	−0.485**
RAVLT long	−0.443**	−0.417**	−0.315*	−0.368**	−0.624**	−0.417**
RAVLT recognition	−0.451**	−0.407**	−0.421**	−0.409**	−0.663**	−0.551**
NPI-Q1	–	0.406**	–	–	–	–
NPI-Q2	–	0.361**	–	–	–	–
HAMA	–	–	–	0.411**	–	–
HAMD	–	0.407**	–	0.423**	–	–
ADL	–	0.368**	–	0.300*	0.456**	0.392**

MD, mean diffusion; F, frontal lobe; P, parietal lobe; O, occipital lobe; T, temporal lobe; Pc, precuneus; H, hippocampus; MMSE, mini-mental state examination; MoCA, Montreal cognitive assessment scale; HDSR, Hasegawa dementia scale revised; CDR, clinical dementia rating scale; DST, digit span test; VFT, verbal fluency test; FAB, frontal assessment battery; TMTA, trail making test A; RAVLT, Rey auditory verbal learning test (immediate recall/short-term delayed recall/long-term delayed recall/recognition); NPI-Q, neuropsychiatric inventory-questionnaire (NPI-Q1: the score of patient, NPI-Q2: the score of caregiver); HAMA, Hamilton anxiety scale; HAMD, Hamilton depression scale; ADL, activity of daily living scale. *p < 0.05, **p < 0.01. Bold data (0.6–0.8), indicating high-intensity correlation.

**TABLE 7:** Pearson’s correlation analysis between MD and neuropsychological test.

MD	SCC	GCC	PIC	LN	CR	CS
MMSE	−0.314*	−0.357**	−0.378**	–	−0.370**	−0.479**
MoCA	–	−0.418**	−0.307*	–	−0.361**	−0.477**
HDSR	−0.262*	−0.397**	−0.396**	–	−0.391**	−0.488**
CDR	–	0.298*	0.333**	–	0.347**	0.512**
DST	−0.254*	−0.500**	–	–	–	−0.341**
VFT	–	−0.515**	–	–	−0.385**	−0.401**
FAB	–	−0.350**	−0.325*	–	−0.381**	−0.433**
TMTA	–	0.356**	0.358**	0.434**	–	0.362**
RAVLT immediate	−0.281*	−0.383**	−0.299*	–	−0.499**	−0.549**
RAVLT short	−0.273*	−0.382**	–	–	−0.473**	−0.516**
RAVLT long	–	−0.385**	–	–	−0.483**	−0.489**
RAVLT recognition	–	−0.446**	−0.271*	–	−0.467**	−0.480**
NPI-Q1	–	–	–	0.319*	0.326*	–
NPI-Q2	–	–	–	0.281*	0.306*	–
HAMA	–	0.394**	–	–	–	–
HAMD	–	0.401**	–	–	–	–
ADL	–	–	–	–	0.380**	0.381**

MD, mean diffusion; SCC, splenium of corpus callosum; GCC, genu of corpus callosum; PIC, posterior limb of internal capsule; LN, lenticular nucleus; CR, corona radiate; CS, centrum semiovale; MMSE, mini-mental state examination; MoCA, Montreal cognitive assessment scale; HDSR, Hasegawa dementia scale revised; CDR, clinical dementia rating scale; DST digit span test; VFT, verbal fluency test; FAB, frontal assessment battery; TMTA, trail making test A; RAVLT, Rey auditory verbal learning test (immediate recall/short-term delayed recall/long-term delayed recall/recognition); NPI-Q, neuropsychiatric inventory-questionnaire (NPI-Q1: the score of patient, NPI-Q2: the score of caregiver); HAMA, Hamilton anxiety scale; HAMD, Hamilton depression scale; ADL, activity of daily living scale. *p < 0.05, **p < 0.01. Bold data (0.6–0.8), indicating high-intensity correlation.

**TABLE 8:** Pearson’s correlation analysis between MK and neuropsychological test.

MK	F	P	O	T	Pc	H
MMSE	0.439**	–	0.283*	0.633**	0.621**	0.688**
MoCA	0.498**	–	0.303*	0.695**	0.573**	0.715**
HDSR	0.467**	–	0.287*	0.680**	0.611**	0.751**
CDR	−0.358**	–	–	−0.632**	−0.585**	−0.716**
DST	0.301*	–	–	0.506**	0.329**	0.499**
VFT	0.365**	–	–	0.556**	0.456**	0.536**
FAB	0.500**	–	–	0.599**	0.581**	0.666**
TMTA	−0.454**	–	–	−0.514**	−0.477**	−0.532**
RAVLT immediate	0.475**	–	0.291*	0.685**	0.621**	0.700**
RAVLT short	0.496**	–	0.253*	0.719**	0.584**	0.705**
RAVLT long	0.414**	–	0.285*	0.689**	0.519**	0.676**
RAVLT recognition	0.457**	–	–	0.680**	0.572**	0.714**
NPI-Q1	−0.314*	−0.258*	–	−0.382**	–	−0.401**
NPI-Q2	−0.306*	–	–	−0.373**	−0.270*	−0.401**
HAMA	–	–	–	−0.349**	–	−0.267*
HAMD	–	–	–	−0.419**	–	−0.363**
ADL	−0.361**	–	–	−0.530**	−0.490**	−0.560**

MK, mean kurtosis; F, frontal lobe; P, parietal lobe; O, occipital lobe; T, temporal lobe; Pc, precuneus; H, hippocampus; MMSE, mini-mental state examination; MoCA, Montreal cognitive assessment scale; HDSR, Hasegawa dementia scale revised; CDR, clinical dementia rating scale; DST, digit span test; VFT, verbal fluency test; FAB, frontal assessment battery; TMTA, trail making test A; RAVLT, Rey auditory verbal learning test (immediate recall/short-term delayed recall/long-term delayed recall/recognition); NPI-Q, neuropsychiatric inventory-questionnaire (NPI-Q1: the score of patient, NPI-Q2: the score of caregiver); HAMA, Hamilton anxiety scale; HAMD, Hamilton depression scale; ADL, activity of daily living scale. *p < 0.05, **p < 0.01. Bold data (0.6–0.8), indicating high-intensity correlation.

**TABLE 9:** Pearson’s correlation analysis between MK and neuropsychological test.

MK	SCC	GCC	PIC	LN	CR	CS
MMSE	0.412**	0.545**	0.441**	–	0.501**	0.492**
MoCA	0.491**	0.535**	0.448**	–	0.519**	0.452**
HDSR	0.437**	0.574**	0.466**	–	0.546**	0.555**
CDR	−0.400**	−0.511**	−0.452**	–	−0.446**	−0.521**
DST	0.450**	0.336**	0.284*	–	0.334**	0.300*
VFT	0.366**	0.382**	0.312*	–	0.499**	0.342**
FAB	0.343**	0.476**	0.431**	–	0.548**	0.452**
TMTA	−0.379**	−0.378**	−0.420**	–	−0.534**	−0.396**
RAVLT immediate	0.429**	0.580**	0.390**	–	0.510**	0.519**
RAVLT short	0.461**	0.552**	0.290*	–	0.454**	0.453**
RAVLT long	0.417**	0.555**	0.262*	–	0.403**	0.426**
RAVLT recognition	0.444**	0.526**	0.374**	–	0.570**	0.533**
NPI-Q1	−0.262*	–	−0.277*	–	−0.477**	–
NPI-Q2	–	–	−0.322*	–	−0.489**	−0.254*
HAMA	–	–	–	–	−0.264**	–
HAMD	–	–	–	–	−0.373**	–
ADL	−0.363**	−0.401**	−0.418**	–	−0.560**	−0.464**

MK, mean kurtosis; SCC, splenium of corpus callosum; GCC, genu of corpus callosum; PIC, posterior limb of internal capsule; LN, lenticular nucleus; CR, corona radiate; CS, centrum semiovale; MMSE, mini-mental state examination; MoCA, Montreal cognitive assessment scale; HDSR, Hasegawa dementia scale revised; CDR, clinical dementia rating scale; DST, digit span test; VFT, verbal fluency test; FAB, frontal assessment battery; TMTA, trail making test A; RAVLT, Rey auditory verbal learning test (immediate recall/short-term delayed recall/long-term delayed recall/recognition); NPI-Q, neuropsychiatric inventory-questionnaire (NPI-Q1: the score of patient, NPI-Q2: the score of caregiver); HAMA, Hamilton anxiety scale; HAMD, Hamilton depression scale; ADL, activity of daily living scale. *p < 0.05, **p < 0.01. Bold data (0.6–0.8), indicating high-intensity correlation.

**FIGURE 5:**
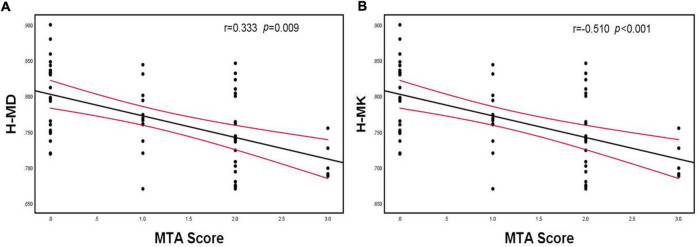
Spearman’s correlation analysis between MD and MK of the hippocampus and MTA scores. **(A)** There was a positive correlation between hippocampus MD and MTA scores, **(B)** There was a negative correlation between hippocampus MK and MTA scores. H-MD, mean diffusivity of hippocampus; H-MK, mean kurtosis of hippocampus; MTA, medial temporal lobe atrophy.

**FIGURE 6:**
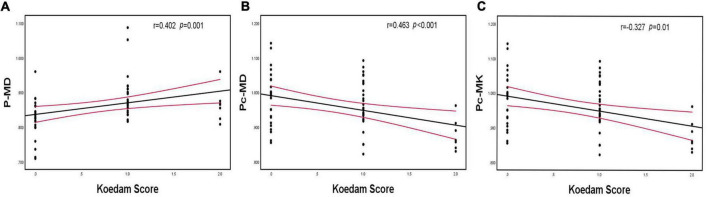
Spearman’s correlation analysis between MD and MK of the parietal lobe and precuneus and Koedam scores. **(A)** Parietal MD was positively correlated with Koedam scores, **(B)** Precuneus MD was positively correlated with Koedam scores, **(C)** Precuneus MK was negatively correlated with Koedam scores. P-MD, mean diffusivity of parietal lobe; Pc-MD, mean diffusivity of precuneus; Pc-MK, mean kurtosis of precuneus.

There was a positive correlation between hippocampus MD and MTA scores and a negative correlation between hippocampus MK and MTA scores. Parietal MD and precuneus MD were positively correlated with Koedam scores, whereas precuneus MK was negatively correlated with Koedam scores (*p* < 0.05; Figures 5, 6).

### Receiver operating characteristic curve analysis of hippocampus mean kurtosis and precuneus mean diffusion

**FIGURE 7:**
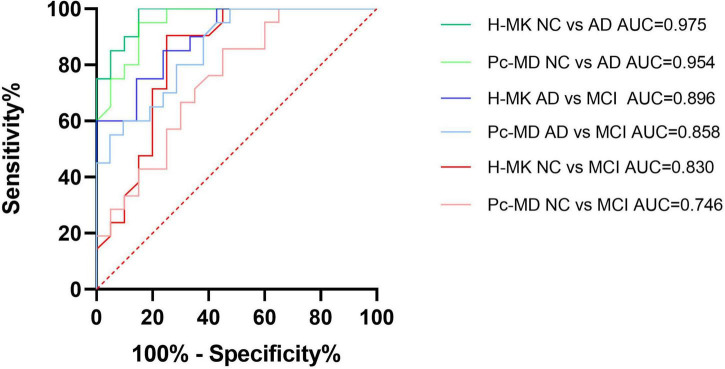
Receiver operating characteristic (ROC) curve analysis of hippocampus MK and precuneus MD. Both parameters could be used to identify any two groups of patients, although hippocampal MK performed better than precuneus MD as reflected by its larger AUC. H-MK, mean kurtosis of hippocampus; Pc-MD, mean diffusivity of precuneus; NC, normal cognition; AD, Alzheimer’s disease; MCI, mild cognitive impairment; AUC, area under the curve.

Table 10 shows the AUC for the ability of hippocampal MK and precuneus MD to distinguish between cognitively normal individuals, patients with MCI, and patients with AD. The diagnostic threshold was determined by the Youden’s index, which is equal to sensitivity plus specificity minus 1, where the value of the variable corresponding to the maximum value of the Youden’s index is the optimal threshold for diagnosis. We demonstrated that both parameters could be used to identify any two groups of patients, although hippocampal MK performed better than precuneus MD as reflected by its larger AUC (Figure 7). When the diagnosis was made according to hippocampus MK, the diagnostic threshold for patients with AD was 0.765, and the diagnostic threshold for patients with MCI was 0.811. When hippocampal MK was selected as the diagnostic threshold, the sensitivity and specificity for AD diagnosis were 85 and 100%, respectively, and the sensitivity and specificity for MCI diagnosis were 75 and 90.5%, respectively.

**TABLE 10:** Coordinate points of receiver operating characteristic (ROC) curve.

Parameter	AUC	Diagnostic threshold	Sensitivity	Specificity
H-MK (NC vs. AD)	0.975	0.765	0.850	1.000
Pc-MD (NC vs. AD)	0.954	0.931	0.950	0.850
H-MK (NC vs. MCI)	0.830	0.811	0.750	0.905
Pc-MD (NC vs. MCI)	0.746	0.864	0.857	0.550
H-MK (AD vs. MCI)	0.896	0.747	0.762	0.850
Pc-MD (AD vs. MCI)	0.858	0.906	1.000	0.524

AUC, area under the curve; H-MK, mean kurtosis of hippocampus; Pc-MD, mean diffusion of precuneus; NC, normal cognition; AD, Alzheimer’s disease; MCI, mild cognitive impairment.

## Discussion

Results showed that FA of the temporal lobe and centrum semiovale was highest in the normal cognition group, lower in the MCI group, and lowest in the AD group. MD of the frontal lobe, parietal lobe, occipital lobe, temporal lobe, precuneus, hippocampus, splenium of the corpus callosum, genu of the corpus callosum, corona radiata, and centrum semiovale was highest in the AD group, followed by the MCI and normal cognition group. MK of the frontal lobe, temporal lobe, precuneus, hippocampus, splenium of the corpus callosum, genu of the corpus callosum, and centrum semiovale was the highest in the normal cognition group, lower in the MCI group, and the lowest in the AD group, which is consistent with the patterns of FA, MD, and MK observed in previous studies (Gong et al., 2013; Struyfs et al., 2015). Moreover, it was clear that MD and MK were more sensitive than FA to detecting differences between the MCI/AD group and the normal cognition group, which was also in agreement with previous studies (Struyfs et al., 2015). MD is the average diffusion coefficient in all diffusion tensor directions and represents the diffusion amplitude of the random motion of water molecules; thus, it is related to tissue integrity. MK represents the average kurtosis in all diffusion directions, where kurtosis describes the peak value of the probability of the water distribution. The higher the kurtosis, the more the water molecule diffusion deviates from the Gaussian distribution, indicating that the diffusion environment is more restrictive. Conversely, reduced kurtosis indicates decreased structural complexity or heterogeneity (Arab et al., 2018; Andica et al., 2019). During the progression of AD, neuronal degeneration, atrophy, and apoptosis lead to an increase in extracellular diffusion space and a decrease in the complexity of brain tissue (Wang et al., 2015). The high MD and low MK in our AD group echo the above changes. Thus, DKI parameters can effectively characterize the changes in the brain pathological conditions of patients with AD.

We performed both ANOVA and ANCOVA on DKI parameters among those three groups. Results showed that the significance of several parameters changed due to age and sex, whereas the key parameters of our research, hippocampal MK and precuneus MD were relatively stable. We found no significant differences in factors such as age, sex, educational level, history of hypertension, history of diabetes, or history of smoking among the three groups, which indicated a balance among the groups, and this might be the reason why the significance of the other parameters was less related. In conclusion, it is of much importance to include age and sex as confounding factors to ensure the quality and reliability of the study. In addition, we carried out FDR correction for the comparisons of DKI parameters to reduce the probability of type 1 errors. Consistent with the results of ANOVA, MD and MK were found to be the most prominent parameters in the process of comparing differences among the three groups; therefore, we selected MD and MK for follow-up analysis.

Studies on the correlation between DKI parameters and neuropsychological scores have been conducted previously, most of which were correlation studies using the MMSE. Diffusion parameters were shown to be negatively correlated with MMSE scores, whereas kurtosis parameters were shown to positively correlate with MMSE scores (Gong et al., 2013). In addition, Li et al. (2022) reported a positive correlation between MK and MoCA scores. Tu et al. (2021) reported a negative correlation between MK and CDR scores, and Yang et al. (2021) found a positive correlation between MK and RAVLT scores. However, the current study is the first to conduct a comprehensive correlation analysis between DKI parameters and neuropsychological test scores. The most sensitive parameters of MD and MK as revealed by the DKI parameter analysis were used to examine associations with comprehensive cognitive scale scores and other scale scores for various cognitive domains. Precuneus MD, temporal MK, hippocampal MK, and precuneus MK were found to be highly correlated with neuropsychological test scores. These brain regions are consistent with the main pathological areas of AD, which suggests that the cognitive decline of patients with AD is due to changes in the complexity of diseased brain tissue and the structure of extracellular spaces. It is worth noting that although the FAB score, which reflects the state of the frontal lobe, was negatively correlated with MD of the frontal lobe and positively correlated with MK of the frontal lobe, the correlation was not particularly strong and lower than that with other brain regions, which may be because pathological changes in AD occur predominantly in the medial temporal lobe. It would be of interest to examine correlations between DKI parameters and the FAB score in patients with frontotemporal lobe dementia.

Common imaging examination methods for AD are positron emission tomography and MRI, which include T1, T2, T2-FLAIR, arterial spin labeling (ASL), DTI, and DKI. Imaging biomarkers have the advantages of being non-invasive, timely, and economical, and are thus the preferred examination method of clinicians. Clinicians can observe brain atrophy in T1, T2, and T2-FLAIR imaging scans, evaluate cerebral blood flow (CBF) in ASL images and quantify brain tissue information using DTI and DKI techniques. DKI technology fully exploits the non-Gaussian diffusion of water molecules to accurately estimate the attenuation of the diffusion-weighted signal and introduce the dimensionless measure of kurtosis to quantify the level of non-Gaussian diffusion. The formula takes into account all types of tissues by using a fourth-order three-dimensional tensor model and provides insight into the changes in microstructure, which may potentially be used as non-invasive biomarkers for AD (Arab et al., 2018; Cheng et al., 2018). Numerous studies comparing DKI and DKI-derived diffusion parameters have shown that kurtosis has a higher diagnostic value than diffusivity (Gong et al., 2013; Yuan et al., 2016; Cheng et al., 2018). Furthermore, Taha et al. (2022) studied the diffusion parameters derived from both DKI and DTI techniques and found that the diffusivity values calculated by DKI were 15–20% higher than those calculated by DTI. Compared with DTI-derived diffusion parameters, DKI-derived diffusion parameters could identify more areas of white matter microstructural changes and favorably reduce the dependence on *b*-values (Giannelli et al., 2013; Wei et al., 2021). Yang et al. (2021) compared the significance of DKI parameters and CBF measured using ASL and found that MK could be used as a potential neuroimaging biomarker and is more sensitive than CBF in the early stage of AD. Thus, we analyzed the correlations between brain atrophy and DKI parameters.

Observing the degree of the medial temporal lobe and posterior atrophy to support the diagnosis of AD is the main purpose of current routine MRI examinations (Lehmann et al., 2012). Wang et al. (2015) analyzed hippocampal volume and DKI parameters and found that DKI parameters are more sensitive than volume for diagnosing MCI and AD. However, studies on the association between brain atrophy scores and DKI parameters have not yet been conducted. Thus, we first compared differences in brain atrophy scores and DKI parameters between groups and found that Dr, FAK, MK, and Kr of the hippocampus and MD and Da of the precuneus differed significantly between the MCI and normal cognition groups, whereas these group differences were not observed in the brain atrophy scores. This confirmed that the changes in the pathological microstructure of patients with AD precede the changes in the macroscopic volume, and DKI parameters can capture these changes acutely. Moreover, the follow-up correlation analysis revealed that the MTA score was most strongly correlated with hippocampal MK, and the Koedam score was most strongly correlated with precuneus MD, which indicated that hippocampal MK and precuneus MD quantitatively reflected brain atrophy scores.

The above analysis of the neuropsychological test and brain atrophy scores showed that hippocampal MK and precuneus MD are the two most sensitive parameters for characterizing cognitive impairment and structural changes and may be used as candidate parameters for diagnosing AD and MCI. The subsequent ROC curve analysis showed that the diagnostic performance of hippocampal MK was better than that of precuneus MD when distinguishing between any two of the groups, and the specificity and sensitivity were more balanced. It is well established that brain atrophy in patients with AD occurs initially in the medial temporal lobe and gradually spreads with the progression of the disease toward the parietal and frontal lobes (Whitwell et al., 2007), which may explain why the diagnostic performance of hippocampal MK was slightly better than that of precuneus MD. Because our study required patients to complete numerous neuropsychological tests and imaging examinations, patients with severe symptoms could not participate; therefore, we could not determine whether precuneus MD has a higher diagnostic value for severe AD. This will require further verification in future studies.

Common biomarkers like amyloid-β (Aβ), total tau (T-tau), and phosphorylated tau (P-tau) reflect the pathological condition of AD and contribute to the diagnosis in the preclinical stage (Blennow and Zetterberg, 2018). Struyfs et al. (2015) analyzed the Aβ1-42, T-tau, and P-tau levels in patients with MCI and AD, but did not compare them with DKI parameters. In other diseases, Lo et al. (2022) studied the correlation of blood tau and Aβ1-42 with DKI parameters in patients with fibromyalgia, but up to date, no one has compared AD pathological biomarkers with DKI. Our study was originally conceived to include *in vitro* pathological studies of AD, but few patients and their families accepted it because of its high cost and invasiveness. On the contrary, DKI technology showing non-invasive, timeliness, and economic advantages would be a promising diagnostic method of AD.

### Limitations

The sample size of our research was limited and the sensitivity of the diagnosis based on hippocampal MK did not reach the same sensitivity as the gold standard. Furthermore, Hyatt et al. (2020) suggested that intracranial volume should be considered a confounding factor; thus, the measurement of intracranial volume needs to be carried out for further study on its relationship with DKI. Nevertheless, DKI technology still has several shortcomings. For example, when the *b*-value changes, all parameter values will change accordingly (Szczepankiewicz et al., 2013; Shahim et al., 2017). Moreover, the present studies are all single-center clinical studies, and the scanning protocols of each center lack consistency. A multi-center, randomized double-blind study needs to be conducted to determine optimal MRI settings for developing an optimal method for non-invasive diagnostic imaging. This will facilitate the application of DKI for patients with AD in routine clinical practice. Last but not the least, DKI needs to be combined with conventional MRI to rule out other diseases.

## Conclusion

Diffusion kurtosis imaging technology can not only detect differences in the microstructure of brain tissue in AD, MCI, and normal cognition but also make up for the deficiencies of brain atrophy scores by detecting the pathological changes in the microstructure of patients with AD before the appearance of macroscopic atrophy. MD and MK of the AD pathological areas were correlated with numerous neuropsychological scale scores, which reflected the severity of cognitive impairment. In particular, hippocampal MK may be used as a sensitive parameter map for evaluating AD and MCI.

## Data availability statement

The raw data supporting the conclusions of this article will be made available by the authors, without undue reservation.

## Ethics statement

The studies involving human participants were reviewed and approved by the Ethics Committee of Second Affiliated Hospital of Dalian Medical University. The patients/participants provided their written informed consent to participate in this study. Written informed consent was obtained from the individual(s) for the publication of any potentially identifiable images or data included in this article.

## Author contributions

YGe and CY conceptualized this study and designed this project. XiC and PW performed the major procedures, wrote the manuscript, revised the manuscript, and approved the final manuscript. XiC, PW, HY, LF, and YD contributed to the data collection. XuC, ZW, CT, YM, YF, and YGu assisted in the analysis of the data. All authors contributed to the article and approved the submitted version.

## Footnotes

1
http://www.fmrib.ox.ac.uk/fsl

2
http://www.mccauslandcenter.sc.edu/mricro/mricron

3
http://www.nitrc.org/projects/dke

## References

[B1] Abdalla G. Dixon L. Sanverdi E. Machado P. M. Kwong J. S. W. Panovska-Griffiths J. (2020). The Diagnostic Role of Diffusional Kurtosis Imaging in Glioma Grading and Differentiation of Gliomas from Other Intra-Axial Brain Tumours: A Systematic Review with Critical Appraisal and Meta-Analysis. *Neuroradiology* 62 791–802. 10.1007/s00234-020-02425-9 32367349 PMC7311378

[B2] Abdelnour C. van Steenoven I. Londos E. Blanc F. Auestad B. Kramberger M. G. (2016). Alzheimer’s Disease Cerebrospinal Fluid Biomarkers Predict Cognitive Decline in Lewy Body Dementia. *Mov. Disord.* 31 1203–1208. 10.1002/mds.26668 27296778

[B3] Albert M. S. DeKosky S. T. Dickson D. Dubois B. Feldman H. H. Fox N. C. (2011). The Diagnosis of Mild Cognitive Impairment due to Alzheimer’s Disease: Recommendations from The National Institute on Aging-Alzheimer’s Association Workgroups on Diagnostic Guidelines for Alzheimer’s Disease. *Alzheimers Dement.* 7 270–279. 10.1016/j.jalz.2011.03.008 21514249 PMC3312027

[B4] Alzheimer’s Association. (2020). 2020 Alzheimer’s Disease Facts and Figures. *Alzheimers Dement.* 16 391–460. 10.1002/alz.12068 32157811

[B5] Andica C. Kamagata K. Hatano T. Saito Y. Ogaki K. Hattori N. (2019). MR Biomarkers of Degenerative Brain Disorders Derived From Diffusion Imaging. *J. Magn. Reason. Imaging* 52 1620–1636. 10.1002/jmri.27019 31837086 PMC7754336

[B6] Arab A. Wojna-Pelczar A. Khairnar A. Szabó N. Ruda-Kucerova J. (2018). Principles of Diffusion Kurtosis Imaging and its Role in Early Diagnosis of Neurodegenerative Disorders. *Brain Res. Bull.* 139 91–98. 10.1016/j.brainresbull.2018.01.015 29378223

[B7] Babulal G. M. Quiroz Y. T. Albensi B. C. Arenaza-Urquijo E. Astell A. J. Babiloni C. (2019). Perspectives on Ethnic and Racial Disparities in Alzheimer’s Disease and Related Dementias: Update and Areas of Immediate Need. *Alzheimers Dement.* 15 292–312. 10.1016/j.jalz.2018.09.009 30555031 PMC6368893

[B8] Blennow K. Zetterberg H. (2018). Biomarkers for Alzheimer’s Disease: Current Status and Prospects for the Future. *J. Intern. Med.* 284 643–663. 10.1111/joim.12816 30051512

[B9] Bonilha L. Lee C. Y. Jensen J. H. Tabesh A. Spampinato M. V. Edwards J. C. (2015). Altered Microstructure in Temporal Lobe Epilepsy: A Diffusional Kurtosis Imaging Study. *AJNR Am. J. Neuroradiol.* 36 719–724. 10.3174/ajnr.A4185 25500311 PMC5559661

[B10] Chen Y. Sha M. Zhao X. Ma J. Ni H. Gao W. (2017). Automated Detection of Pathologic White Matter Alterations in Alzheimer’s Disease Using Combined Diffusivity and Kurtosis Method. *Psychiatry Res. Neuroimaging* 264 35–45. 10.1016/j.pscychresns.2017.04.004 28448817

[B11] Cheng J. X. Zhang H. Y. Peng Z. K. Xu Y. Tang H. Wu J. T. (2018). Divergent Topological Networks in Alzheimer’s Disease: A Diffusion Kurtosis Imaging Analysis. *Transl. Neurodegener.* 7:10. 10.1186/s40035-018-0115-y 29719719 PMC5921324

[B12] Falangola M. F. Jensen J. H. Tabesh A. Hu C. Deardorff R. L. Babb J. S. (2013). Non-Gaussian Diffusion MRI Assessment of Brain Microstructure in Mild Cognitive Impairment and Alzheimer’s Disease. *Magn. Reason. Imaging* 31 840–846. 10.1016/j.mri.2013.02.008 23602730 PMC5347444

[B13] Giannelli M. Diciotti S. Guerrisi M. Traino A. C. Mascalchi M. Tessa C. (2013). On the Estimation of Conventional DTI-Derived Indices by Fitting the Non-Gaussian DKI Model to Diffusion-Weighted Imaging Datasets. *Neuroradiology* 55 1423–1424. 10.1007/s00234-013-1271-5 24005831

[B14] Gong N. J. Chan C. C. Leung L. M. Wong C. S. Dibb R. Liu C. (2017). Differential Microstructural and Morphological Abnormalities in Mild Cognitive Impairment and Alzheimer’s Disease: Evidence from Cortical and Deep Gray Matter. *Hum. Brain Mapp.* 38 2495–2508. 10.1002/hbm.23535 28176436 PMC6867186

[B15] Gong N. J. Wong C. S. Chan C. C. Leung L. M. Chu Y. C. (2013). Correlations Between Microstructural Alterations and Severity of Cognitive Deficiency in Alzheimer’s Disease and Mild Cognitive Impairment: A Diffusional Kurtosis Imaging Study. *Magn. Reason. Imaging* 31 688–694. 10.1016/j.mri.2012.10.027 23347602

[B16] Hui E. S. Fieremans E. Jensen J. H. Tabesh A. Feng W. Bonilha L. (2012). Stroke Assessment with Diffusional Kurtosis Imaging. *Stroke* 43 2968–2973. 10.1161/STROKEAHA.112.657742 22933581 PMC3479373

[B17] Hyatt C. S. Owens M. M. Crowe M. L. Carter N. T. Lynam D. R. Miller J. D. (2020). The Quandary of Covarying: A Brief Review and Empirical Examination of Covariate Use in Structural Neuroimaging Studies on Psychological Variables. *Neuroimage* 205:116225. 10.1016/j.neuroimage.2019.116225 31568872

[B18] Jensen J. H. Helpern J. A. (2010). MRI Quantification of Non-Gaussian Water Diffusion by Kurtosis Analysis. *NMR Biomed.* 23 698–710. 10.1002/nbm.1518 20632416 PMC2997680

[B19] Kritsilis M. Rizou S. Koutsoudaki P. N. Evangelou K. Gorgoulis V. G. Papadopoulos D. (2018). Ageing, Cellular Senescence and Neurodegenerative Disease. *Int. J. Mol. Sci.* 19:2937. 10.3390/ijms19102937 30261683 PMC6213570

[B20] Lane C. A. Hardy J. Schott J. M. (2018). Alzheimer’s Disease. *Eur. J. Neurol.* 25 59–70. 10.1111/ene.13439 28872215

[B21] Lehmann M. Koedam E. L. Barnes J. Bartlett J. W. Ryan N. S. Pijnenburg Y. A. (2012). Posterior Cerebral Atrophy in the Absence of Medial Temporal Lobe Atrophy in Pathologically-Confirmed Alzheimer’s Disease. *Neurobiol. Aging* 33:627.e1–627.e12. 10.1016/j.neurobiolaging.2011.04.003 21596458 PMC3657170

[B22] Li T. Zhang Y. Fu X. Zhang X. Luo Y. Ni H. (2022). Microstructural White Matter Alterations in Alzheimer’s Disease and Amnestic Mild Cognitive Impairment and its Diagnostic Value Based on Diffusion Kurtosis Imaging: A Tract-Based Spatial Statistics Study. *Brain Imaging Behav.* 16 31–42. 10.1007/s11682-021-00474-z 33895943

[B23] Lo Y. C. Li T. J. T. Lin T. C. Chen Y. Y. Kang J. H. (2022). Microstructural Evidence of Neuroinflammation for Psychological Symptoms and Pain in Patients with Fibromyalgia. *J. Rheumatol.* 49, 942–947. 10.3899/jrheum.211170 35501148

[B24] Mantzavinos V. Alexiou A. (2017). Biomarkers for Alzheimer’s Disease Diagnosis. *Curr. Alzheimer Res.* 14 1149–1154. 10.2174/1567205014666170203125942 28164766 PMC5684784

[B25] Petersen R. C. (2004). Mild Cognitive Impairment as a Diagnostic Entity. *J. Intern. Med.* 256 183–194. 10.1111/j.1365-2796.2004.01388.x 15324362

[B26] Roberts R. Knopman D. S. (2013). Classification and Epidemiology of MCI. *Clin. Geriatr. Med.* 29 753–772. 10.1016/j.cger.2013.07.003 24094295 PMC3821397

[B27] Rusek M. Pluta R. Ułamek-Kozioł M. Czuczwar S. J. (2019). Ketogenic Diet in Alzheimer’s Disease. *Int. J. Mol. Sci.* 20:3892. 10.3390/ijms20163892 31405021 PMC6720297

[B28] Shahim P. Holleran L. Kim J. H. Brody D. L. (2017). Test-Retest Reliability of High Spatial Resolution Diffusion Tensor and Diffusion Kurtosis Imaging. *Sci. Rep.* 7:11141. 10.1038/s41598-017-11747-3 28894296 PMC5593980

[B29] Song G. P. Yao T. T. Wang D. Li Y. H. (2019). Differentiating Between Alzheimer’s Disease, Amnestic Mild Cognitive Impairment, and Normal Aging via Diffusion Kurtosis Imaging. *Neural Regen. Res.* 14 2141–2146. 10.4103/1673-5374.262594 31397353 PMC6788254

[B30] Soria L. J. A. González H. M. Léger G. C. (2019). Alzheimer’s Disease. *Handb. Clin. Neurol.* 167 231–255. 10.1016/B978-0-12-804766-8.00013-3 31753135

[B31] Struyfs H. Van H. W. Veraart J. Sijbers J. Slaets S. De Belder M. (2015). Diffusion Kurtosis Imaging: A Possible MRI Biomarker for AD Diagnosis? *J. Alzheimers Dis.* 48 937–948. 10.3233/JAD-150253 26444762 PMC4927852

[B32] Szczepankiewicz F. Lätt J. Wirestam R. Leemans A. Sundgren P. van Westen D. (2013). Variability in Diffusion Kurtosis Imaging: Impact on Study Design, Statistical Power and Interpretation. *Neuroimage* 76 145–154. 10.1016/j.neuroimage.2013.02.078 23507377

[B33] Taha H. T. Chad J. A. Chen J. J. (2022). DKI Enhances the Sensitivity and Interpretability of Age-Related DTI Patterns in the White Matter of UK Biobank Participants. *Neurobiol. Aging* 115 39–49. 10.1016/j.neurobiolaging.2022.03.008 35468551

[B34] Tu M. C. Huang S. M. Hsu Y. H. Yang J. J. Lin C. Y. Kuo L. W. (2021). Discriminating Subcortical Ischemic Vascular Disease and Alzheimer’s Disease by Diffusion Kurtosis Imaging in Segregated Thalamic Regions. *Hum. Brain. Mapp.* 42 2018–2031. 10.1002/hbm.25342 33416206 PMC8046043

[B35] Wang D. Guo Z. H. Liu X. H. Li Y. H. Wang H. (2015). Examination of Hippocampal Differences Between Alzheimer’s Disease, Amnestic Mild Cognitive Impairment and Normal Aging: Diffusion Kurtosis. *Curr. Alzheimer Res.* 12 80–87. 10.2174/1567205012666141218142422 25523422

[B36] Wei Y. Wang C. Liu J. Miao P. Wei S. Wang Y. (2021). Widespread White Matter Microstructure Alterations Based on Diffusion Tensor Imaging and Diffusion Kurtosis Imaging in Patients With Pontine Infarction. *Front. Aging Neurosci.* 13:758236. 10.3389/fnagi.2021.758236 34975452 PMC8714656

[B37] Weller J. Budson A. (2018). Current Understanding of Alzheimer’s Disease Diagnosis and Treatment. *F1000Res.* 7 1161–1169. 10.12688/f1000research.14506.1 30135715 PMC6073093

[B38] Whitwell J. L. Przybelski S. A. Weigand S. D. Knopman D. S. Boeve B. F. Petersen R. C. (2007). 3D Maps from Multiple MRI Illustrate Changing Atrophy Patterns as Subjects Progress from Mild Cognitive Impairment to Alzheimer’s Disease. *Brain* 130 1777–1786. 10.1093/brain/awm112 17533169 PMC2752411

[B39] Yang Z. Rong Y. Cao Z. Wu Y. Zhao X. Xie Q. (2021). Microstructural and Cerebral Blood Flow Abnormalities in Subjective Cognitive Decline Plus: Diffusional Kurtosis Imaging and Three-Dimensional Arterial Spin Labeling Study. *Front. Aging Neurosci.* 13:625843. 10.3389/fnagi.2021.625843 33597860 PMC7882515

[B40] Yuan L. Sun M. Chen Y. Long M. Zhao X. Yin J. (2016). Non-Gaussian Diffusion Alterations on Diffusion Kurtosis Imaging in Patients with Early Alzheimer’s Disease. *Neurosci. Lett.* 616 11–18. 10.1016/j.neulet.2016.01.021 26797581

[B41] Zhang G. Zhang Y. Zhang C. Wang Y. Ma G. Nie K. (2015). Diffusion Kurtosis Imaging of Substantia Nigra is a Sensitive Method for Early Diagnosis and Disease Evaluation in Parkinson’s Disease. *Parkinsons Dis.* 2015:207624. 10.1155/2015/207624 26770867 PMC4681830

