# Supplementary material for: Comparison of brain microstructure alterations on diffusion kurtosis imaging among Alzheimer’s disease, mild cognitive impairment, and cognitively normal individuals
Source: Front Aging Neurosci. 2022 Aug 12;14:919143. doi: 10.3389/fnagi.2022.919143 (PMC9416000; doi:10.3389/fnagi.2022.919143)
Supplement: Supplementary file 1 [file Data_Sheet_1.docx]

**Table S1** Mean (± standard deviation) parameters for each ROI for each study group in both one-way analysis of variance (ANOVA) and analysis of covariance (ANCOVA)

|  | NC | MCI | AD | F-ANOVA | *P*-ANOVA | F-ANCOVA | *P*-ANCOVA |
| --- | --- | --- | --- | --- | --- | --- | --- |
| T-FA | 0.260 ± 0.094 | 0.200 ± 0.077* | 0.164 ± 0.062* | 7.452 | 0.001 | 3.525 | 0.036 |
| CS-FA | 0.392 ± 0.102 | 0.354 ± 0.111 | 0.267 ± 0.097*# | 7.666 | 0.001 | 6.779 | 0.002 |
| F-MD | 0.824 ± 0.048 | 0.839 ± 0.044 | 0.899 ± 0.075*# | 9.829 | < 0.001 | 8.692 | 0.001 |
| P-MD | 0.826 ± 0.048 | 0.860 ± 0.050* | 0.897 ± 0.073*# | 7.549 | 0.001 | 5.575 | 0.006 |
| O-MD | 0.840 ± 0.041 | 0.851 ± 0.055 | 0.901 ± 0.083*# | 5.485 | 0.007 | 3.600 | 0.034 |
| T-MD | 0.943 ± 0.121 | 0.972 ± 0.117 | 1.054 ± 0.090*# | 5.448 | 0.007 | 2.900 | 0.064 |
| Pc-MD | 0.866 ± 0.060 | 0.918 ± 0.055* | 1.028 ± 0.087*# | 29.579 | < 0.001 | 23.559 | < 0.001 |
| H-MD | 0.991 ± 0.113 | 1.044 ± 0.117 | 1.224 ± 0.208*# | 13.016 | < 0.001 | 7.934 | 0.001 |
| SCC-MD | 0.927 ± 0.116 | 0.959 ± 0.133 | 1.029 ± 0.123*# | 3.536 | 0.036 | 7.830 | 0.001 |
| GCC-MD | 0.940 ± 0.080 | 1.038 ± 0.159* | 1.103 ± 0.144* | 7.632 | 0.001 | 6.006 | 0.004 |
| PIC-MD | 0.853 ± 0.061 | 0.834 ± 0.076 | 0.904 ± 0.070*# | 5.559 | 0.006 | 4.461 | 0.016 |
| CR-MD | 0.823 ± 0.040 | 0.827 ± 0.067 | 0.888 ± 0.064*# | 8.036 | 0.001 | 6.479 | 0.003 |
| CS-MD | 0.801 ± 0.038 | 0.815 ± 0.055 | 0.874 ± 0.064*# | 10.564 | < 0.001 | 7.159 | 0.002 |
| Pc-Da | 1.100 ± 0.102 | 1.219 ± 0.103* | 1.361 ± 0.193*# | 17.823 | < 0.001 | 19.189 | < 0.001 |
| H-Da | 1.284 ± 0.119 | 1.318 ± 0.137 | 1.486 ± 0.187*# | 10.396 | < 0.001 | 8.776 | 0.001 |
| SCC-Da | 1.702 ± 0.226 | 1.773 ± 0.259 | 1.940 ± 0.378* | 3.464 | 0.038 | 3.766 | 0.029 |
| PIC-Da | 1.458 ± 0.187 | 1.505 ± 0.201 | 1.621 ± 0.221* | 3.394 | 0.04 | 2.608 | 0.083 |
| LN-Da | 1.068 ± 0.198 | 1.193 ± 0.248 | 1.271 ± 0.256* | 3.763 | 0.029 | 2.768 | 0.072 |
| F-Dr | 0.641 ± 0.055 | 0.679 ± 0.070 | 0.700 ± 0.114* | 2.634 | 0.080 | 3.761 | 0.030 |
| P-Dr | 0.600 ± 0.081 | 0.627 ± 0.073 | 0.680 ± 0.099* | 4.676 | 0.013 | 2.164 | 0.125 |
| O-Dr | 0.649 ± 0.069 | 0.641 ± 0.097 | 0.749 ± 0.077*# | 10.869 | < 0.001 | 8.939 | < 0.001 |
| T-Dr | 0.753 ± 0.138 | 0.769 ± 0.135 | 0.922 ± 0.096*# | 11.212 | < 0.001 | 8.194 | 0.001 |
| Pc-Dr | 0.723 ± 0.083 | 0.743 ± 0.097 | 0.816 ± 0.113*# | 4.939 | 0.01 | 2.910 | 0.063 |
| H-Dr | 0.777 ± 0.118 | 0.932 ± 0.184* | 1.099 ± 0.279*# | 12.337 | < 0.001 | 6.706 | 0.003 |
| GCC-Dr | 0.489 ± 0.150 | 0.630 ± 0.155* | 0.649 ± 0.130* | 7.247 | 0.002 | 9.423 | < 0.001 |
| CS-Dr | 0.585 ± 0.092 | 0.609 ± 0.069 | 0.689 ± 0.090*# | 8.316 | 0.001 | 5.426 | 0.007 |
| P-FAK | 0.426 ± 0.099 | 0.376 ± 0.075 | 0.351 ± 0.091* | 3.662 | 0.032 | 2.102 | 0.132 |
| T-FAK | 0.405 ± 0.099 | 0.329 ± 0.098* | 0.295 ± 0.093* | 6.8 | 0.002 | 2.792 | 0.070 |
| H-FAK | 0.346 ± 0.088 | 0.262 ± 0.089* | 0.205 ± 0.057*# | 16.018 | < 0.001 | 11.217 | < 0.001 |
| PIC-FAK | 0.486 ± 0.088 | 0.538 ± 0.067* | 0.510 ± 0.091 | 1.994 | 0.145 | 3.754 | 0.030 |
| CS-FAK | 0.482 ± 0.115 | 0.427 ± 0.091 | 0.375 ± 0.137* | 4.314 | 0.018 | 1.049 | 0.357 |
| F-MK | 1.037 ± 0.061 | 1.000 ± 0.070 | 0.955 ± 0.060*# | 8.27 | 0.001 | 5.669 | 0.006 |
| T-MK | 0.955 ± 0.077 | 0.883 ± 0.040* | 0.806 ± 0.042*# | 36.314 | < 0.001 | 32.049 | < 0.001 |
| Pc-MK | 0.997 ± 0.062 | 0.987 ± 0.079 | 0.895 ± 0.042*# | 16.141 | < 0.001 | 11.752 | < 0.001 |
| H-MK | 0.823 ± 0.042 | 0.773 ± 0.035* | 0.709 ± 0.033*# | 48.486 | < 0.001 | 31.427 | < 0.001 |
| SCC-MK | 1.149 ± 0.052 | 1.086 ± 0.070* | 1.052 ± 0.070* | 11.577 | < 0.001 | 6.900 | 0.002 |
| GCC-MK | 1.046 ± 0.074 | 0.972 ± 0.082* | 0.900 ± 0.074*# | 17.849 | < 0.001 | 20.341 | < 0.001 |
| PIC-MK | 1.207 ± 0.051 | 1.221 ± 0.056 | 1.156 ± 0.069*# | 6.679 | 0.002 | 3.692 | 0.031 |
| CR-MK | 1.165 ± 0.041 | 1.176 ± 0.066 | 1.091 ± 0.053*# | 14.656 | < 0.001 | 10.919 | < 0.001 |
| CS-MK | 1.130 ± 0.054 | 1.130 ± 0.073 | 1.046 ± 0.044*# | 13.933 | < 0.001 | 9.322 | < 0.001 |
| Pc-Ka | 0.858 ± 0.147 | 0.803 ± 0.085 | 0.738 ± 0.083* | 6.071 | 0.004 | 4.815 | 0.012 |
| H-Ka | 0.751 ± 0.071 | 0.735 ± 0.099 | 0.652 ± 0.102*# | 6.674 | 0.002 | 5.562 | 0.006 |
| F-Kr | 1.198 ± 0.201 | 1.169 ± 0.219 | 1.046 ± 0.194* | 3.115 | 0.052 | 3.193 | 0.049 |
| T-Kr | 1.013 ± 0.185 | 0.912 ± 0.126* | 0.815 ± 0.071*# | 10.809 | < 0.001 | 7.808 | 0.001 |
| Pc-Kr | 1.108 ± 0.148 | 1.104 ± 0.233 | 0.941 ± 0.218*# | 4.391 | 0.017 | 2.390 | 0.101 |
| H-Kr | 0.904 ± 0.125 | 0.805 ± 0.090* | 0.713 ± 0.081*# | 18.322 | < 0.001 | 11.663 | < 0.001 |
| GCC-Kr | 1.632 ± 0.402 | 1.370 ± 0.270* | 1.284 ± 0.245* | 6.747 | 0.002 | 10.420 | < 0.001 |
| LN-Kr | 0.812 ± 0.189 | 0.957 ± 0.247* | 1.019 ± 0.243* | 4.355 | 0.017 | 3.636 | 0.033 |

NC: normal cognition; MCI: mild cognitive impairment; AD: Alzheimer’s disease; FA: fractional anisotropy; MD: mean diffusion; Da: axial diffusion; Dr: radial diffusion; FAK: fractional anisotropy of kurtosis; MK: mean kurtosis; Ka: axial kurtosis; Kr: radial kurtosis; F: frontal lobe; P: parietal lobe; O: occipital lobe; T: temporal lobe; Pc: precuneus; H: hippocampus; SCC: splenium of the corpus callosum; GCC: genu of the corpus callosum; PIC: posterior limb of the internal capsule; LN: lenticular nucleus; CR: coronal radiata; CS: centrum semiovale.

**p* < 0.05 vs. NC group in ANOVA, #*p* < 0.05 vs. MCI group in ANOVA; **p* < 0.05 vs. NC group in ANCOVA, #*p* < 0.05 vs. MCI group in ANCOVA; **p* < 0.05 vs. NC group in both analysis, #*p* < 0.05 vs. MCI group in both analysis.

**Table S2** Significant parameters for each ROI for each study group in FDR correction

|  | NC vs. AD | | NC vs. MCI | | AD vs. MCI | |
| --- | --- | --- | --- | --- | --- | --- |
|  | t | *p* (FDR) | t | *p* (FDR) | t | *p* (FDR) |
| T-FA | -3.733 | 0.003* | -2.204 | 0.211 | -1.591 | 0.260 |
| CS-FA | -4.002 | 0.001* | -1.168 | 0.632 | -2.673 | 0.049* |
| F-MD | 3.628 | 0.003* | 0.900 | 0.674 | 3.046 | 0.024* |
| P-MD | 3.486 | 0.004* | 2.063 | 0.229 | 1.814 | 0.191 |
| O-MD | 2.844 | 0.018* | 0.671 | 0.725 | 2.212 | 0.104 |
| T-MD | 3.323 | 0.006* | 0.799 | 0.675 | 2.515 | 0.066 |
| Pc-MD | 6.774 | < 0.001* | 2.774 | 0.079 | 4.775 | 0.001* |
| H-MD | 4.524 | < 0.001* | 1.496 | 0.471 | 3.535 | 0.011* |
| SCC-MD | 2.736 | 0.022* | 0.830 | 0.675 | 1.785 | 0.195 |
| GCC-MD | 4.469 | < 0.001* | 2.519 | 0.125 | 1.402 | 0.317 |
| PIC-MD | 2.366 | 0.047* | -0.868 | 0.674 | 3.005 | 0.024* |
| CR-MD | 3.643 | 0.003* | 0.220 | 0.925 | 2.906 | 0.029* |
| CS-MD | 4.110 | 0.001* | 0.885 | 0.674 | 2.999 | 0.024* |
| Pc-Da | 5.492 | < 0.001* | 3.767 | 0.026* | 3.030 | 0.024* |
| H-Da | 4.173 | 0.001* | 0.855 | 0.674 | 3.376 | 0.014* |
| SCC-Da | 2.507 | 0.035* | 0.972 | 0.674 | 1.716 | 0.219 |
| PIC-Da | 2.587 | 0.030* | 0.798 | 0.675 | 1.808 | 0.191 |
| LN-Da | 2.890 | 0.016* | 1.835 | 0.321 | 1.019 | 0.450 |
| CR-Da | 2.725 | 0.022* | 1.301 | 0.550 | 0.938 | 0.485 |
| P-Dr | 2.820 | 0.019* | 1.137 | 0.632 | 1.941 | 0.157 |
| O-Dr | 4.241 | 0.001* | -0.307 | 0.890 | 3.926 | 0.004* |
| T-Dr | 4.554 | < 0.001* | 0.382 | 0.866 | 4.203 | 0.002* |
| Pc-Dr | 2.970 | 0.014* | 0.706 | 0.715 | 2.236 | 0.102 |
| H-Dr | 4.885 | < 0.001* | 3.253 | 0.053 | 2.343 | 0.082 |
| GCC-Dr | 3.684 | 0.003* | 3.027 | 0.053 | 0.443 | 0.746 |
| CS-Dr | 3.582 | 0.003* | 0.944 | 0.674 | 3.141 | 0.023* |
| P-FAK | -2.483 | 0.036* | -1.804 | 0.327 | -0.956 | 0.479 |
| T-FAK | -3.637 | 0.003* | -2.480 | 0.127 | -1.144 | 0.408 |
| H-FAK | -5.912 | < 0.001* | -3.031 | 0.053 | -2.406 | 0.079 |
| CS-FAK | -2.727 | 0.022* | -1.717 | 0.361 | -1.466 | 0.300 |
| F-MK | -4.084 | < 0.001* | -1.714 | 0.361 | -2.155 | 0.114 |
| T-MK | -7.291 | < 0.001* | -3.615 | 0.026* | -5.246 | < 0.001* |
| Pc-MK | -5.736 | < 0.001* | -0.466 | 0.824 | -4.439 | 0.002* |
| H-MK | -8.193 | < 0.001* | -3.602 | 0.026* | -4.972 | 0.001* |
| SCC-MK | -4.765 | < 0.001* | -3.135 | 0.053 | -1.492 | 0.299 |
| GCC-MK | -6.075 | < 0.001* | -2.970 | 0.053 | -2.892 | 0.029* |
| PIC-MK | -2.526 | 0.035* | 0.764 | 0.684 | -3.143 | 0.023* |
| CR-MK | -4.516 | < 0.001* | 0.592 | 0.774 | -4.328 | 0.002* |
| CS-MK | -4.995 | < 0.001* | -0.033 | 0.976 | -4.219 | 0.002* |
| Pc-Ka | -3.221 | 0.008* | -1.495 | 0.471 | -2.443 | 0.075 |
| H-Ka | -3.538 | 0.003* | -0.581 | 0.774 | -2.649 | 0.049* |
| F-Kr | -2.512 | 0.035* | -0.459 | 0.824 | -1.957 | 0.156 |
| T-Kr | -4.585 | < 0.001* | -2.109 | 0.229 | -3.064 | 0.024* |
| Pc-Kr | -2.917 | 0.016* | -0.079 | 0.976 | -2.372 | 0.079 |
| H-Kr | -5.806 | < 0.001* | -2.968 | 0.053 | -3.434 | 0.013* |
| GCC-Kr | -3.424 | 0.005* | -2.555 | 0.125 | -1.099 | 0.418 |
| LN-Kr | 3.109 | 0.010* | 2.176 | 0.211 | 0.837 | 0.543 |

NC: normal cognition; MCI: mild cognitive impairment; AD: Alzheimer’s disease; FA: fractional anisotropy; MD: mean diffusion; Da: axial diffusion; Dr: radial diffusion; FAK: fractional anisotropy of kurtosis; MK: mean kurtosis; Ka: axial kurtosis; Kr: radial kurtosis; F: frontal lobe; P: parietal lobe; O: occipital lobe; T: temporal lobe; Pc: precuneus; H: hippocampus; SCC: splenium of the corpus callosum; GCC: genu of the corpus callosum; PIC: posterior limb of the internal capsule; LN: lenticular nucleus; CR: coronal radiata; CS: centrum semiovale.

**p* (FDR) < 0.05.
